# Phenotyping and susceptibility of established porcine cells lines to African Swine Fever Virus infection and viral production

**DOI:** 10.1038/s41598-017-09948-x

**Published:** 2017-09-04

**Authors:** Elena G. Sánchez, Elena Riera, Marisa Nogal, Carmina Gallardo, Paloma Fernández, Raquel Bello-Morales, José Antonio López-Guerrero, Carol G. Chitko-McKown, Jürgen A. Richt, Yolanda Revilla

**Affiliations:** 1 Virology Department, Centro Biología Molecular Severo Ochoa, CSIC-UAM, Madrid, Spain; 2 Kansas State University, College of Veterinary Medicine, Center of Excellence for Emerging and Zoonotic Animal Diseases (CEEZAD) Manhattan (KS), Kansas, USA; 3 Molecular Biology Department, Universidad Autónoma de Madrid, Madrid, Spain; 4 USDA, ARS, U.S. Meat Animal Research Center (USMARC), Clay Center, P.O. Box 166, NE68933 Nebraska, USA; 5 European Union Reference laboratory for ASF, CISA-INIA, Madrid, Spain

## Abstract

African swine fever virus (ASFV) is a highly pathogenic, double-stranded DNA virus with a marked tropism for cells of the monocyte-macrophage lineage, affecting swine species and provoking severe economic losses and health threats. In the present study, four established porcine cell lines, IPAM-WT, IPAM-CD163, C∆2+ and WSL, were compared to porcine alveolar macrophage (PAM) in terms of surface marker phenotype, susceptibility to ASFV infection and virus production. The virulent ASFV Armenia/07, E70 or the naturally attenuated NHV/P68 strains were used as viral models. Cells expressed only low levels of specific receptors linked to the monocyte/macrophage lineage, with low levels of infection overall, with the exception of WSL, which showed more efficient production of strain NHV/P68 but not of strains E70 and Armenia/07.

## Introduction

African swine fever virus (ASFV) is the causative agent of African swine fever (ASF), a highly contagious disease affecting different species of swine^1^. Symptoms range from acute fatal haemorrhagic fever to more chronic or unapparent infection depending on the virulence of the isolate^2^. ASFV is endemic in sub-Saharan Africa and Sardinia, but transcontinental transmission in 2007 introduced it into Georgia and Armenia, later spreading to Russia and Ukraine in 2012^3, 4^. ASF causes major economic losses, threatens food security, and limits pig production in affected countries. The fact that no vaccine is currently available makes knowledge and tools against ASFV strong priorities in the veterinary field.

ASFV is an enveloped, double-stranded DNA icosahedral virus with a diameter of 200 nm^5^, formed by several concentric layers. Its genome encodes more than 150 ORFs with functions related to DNA replication, gene transcription and host cell interaction^6–13^. Viral replication is mainly cytoplasmic, taking place around 10–12 h post-infection (hpi) in perinuclear viral factories, although a nuclear step has been reported^14^; gene expression is highly regulated temporally, with four stages of transcription: immediate-early, early, intermediate and late^15, 16^.

In pigs, monocytes and alveolar macrophages are the main targets for ASFV infection^1, 17^, important for viral pathogenesis as these cells play a central role in the immune response through phagocytosis, antigen presentation and cytokine secretion^18, 19^. Porcine alveolar macrophages (PAM) are known to express CD14, SLAII, CD163, CD169, CD203, SWC3 (CD172a) and CD16 receptors^20^. SWC3 and CD14 are specific receptors of the myeloid lineage. The expression of SWC3 occurs in the precursor of myeloid cells and is maintained at all stages of differentiation ^21^; CD14 is expressed on monocytes, tissue macrophages and, at lower levels, on granulocytes^22^. CD203 is also present on thymocytes and in monocytes its expression is increased during their differentiation into macrophages^23, 24^. CD163 is a member of the scavenger receptor cysteine-rich domain family whose expression is restricted to the monocyte/macrophage lineage and is usually employed as a marker for monocytic differentiation and maturation^25, 26^. This molecule acts as a receptor of the hemoglobin/haptoglobin complex, activating a signalling pathway that provokes the production of pro- and anti- inflammatory cytokines^25, 27^. CD163 can also be regulated by lipopolysaccharide (LPS) or interleukin-10 (IL-10)^28^. CD163 plays a fundamental role during the uncoating of the porcine reproductive and respiratory syndrome virus (PRRSV) from endosomes to the cytoplasm^29^. Porcine CD169 or Siglec-1 is a membrane glycoprotein induced by IFN-α and expressed by different populations of tissue macrophages (but not monocytes)^30^. Its function has not yet been determined, although it has recently been suggested as a modulator of inflammatory and immune responses^31^ and phagocytosis through interaction with other receptors^32^. CD169 has also been described as a receptor for PRRSV in an endocytic process mediated by clathrin^33^.

ASFV enters host cells by receptor-mediated endocytosis, which is a pH, temperature, energy and cholesterol-dependent process^34–36^. The first steps of viral internalization involve macropinocytosis and clathrin mechanisms, although the cellular attachment factors and viral ligand are not yet fully understood^35, 37–42^. However, the susceptibility of host cells to ASFV seems to be linked to maturity since *in vitro* maturation of porcine blood monocyte cells (PBMCs) to macrophages, correlating with an up-regulation of CD203 and CD163 expression, has been shown to increase ASFV infection^24, 43^. Nevertheless, the role of CD163 in ASFV infection is controversial since it has been published that the expression of CD163 alone is not enough to increase the susceptibility to the virus in non-permissive cells^44^, and pigs lacking CD163 showed no resistance to infection with the ASFV isolate Georgia 2007/1^45^.

Although the use of primary monocytes or alveolar macrophages for ASFV studies offers obvious advantages in terms of study of virus-host interaction and mimicry of infection *in vivo*, there are several drawbacks to working with primary cultures. These issues of difficult reproducibility, lot-to-lot variation, laborious and costly cell extraction and animal welfare concerns, were partially overcome several years ago by the adaptation of the Ba71 (virulent) strain to grow in Vero cells (Ba71V)^46^. Lately, COS-7 (an SV40 T-antigen-transformed epithelial green monkey cell line), was used as a model for the infection, since it allowed for productive ASFV infection^7, 47^. Nevertheless, the use of a host-derived cell system is needed to maintain a more natural environment for studies of cell-host interaction and immune response. Additionally, the generation of live attenuated vaccines (LAV) requires porcine cell lines that are able to sustain productive ASFV infection.

In the present study, we have characterized four different porcine cell lines, IPAM WT^48^, IPAM-CD163^49^, WSL^50, 51^ and CΔ2+^52^, in terms of their macrophage surface marker phenotype, susceptibility to ASFV infection and virus production capacity. Two different virulent ASFV isolates, Armenia/07 and E70, and the attenuated NHV/P68 were used as viral models, in order to test the effect of virulence on infectivity in porcine cell lines. None of the tested cell lines showed a mature macrophage phenotype. The level of infection and virus production in IPAM-WT and CΔ2+ was much lower than in PAM, whereas WSL was able to sustain ASFV infection albeit with several differences compared with PAM.

## Results

### Characterization of receptors linked to the monocyte/macrophage lineage on IPAM-WT, IPAM-CD163, WSL and CΔ2+ cells

Since the monocyte/macrophage lineage represents the ASFV target cell in pigs, the first objective of our study was to determine the pattern of specific markers associated to this linage on different existent porcine cell lines. Among them, CD14 and SWC3 expression was first examined as markers of myelomonocytic phenotype. Furthermore, since the presence of CD163 and CD169 receptors is linked both to differentiation and maturation state in monocyte/macrophage lineages, and to ASFV susceptibility in PAM^43^, we further analysed the membrane expression of these markers in IPAM-WT, IPAM-CD163, WSL, CΔ2+ and COS-7 cells. SLA-II and SLA-I were also used to show cellular antigen presentation and as a positive control of porcine origin, respectively.

**Figure 1:**
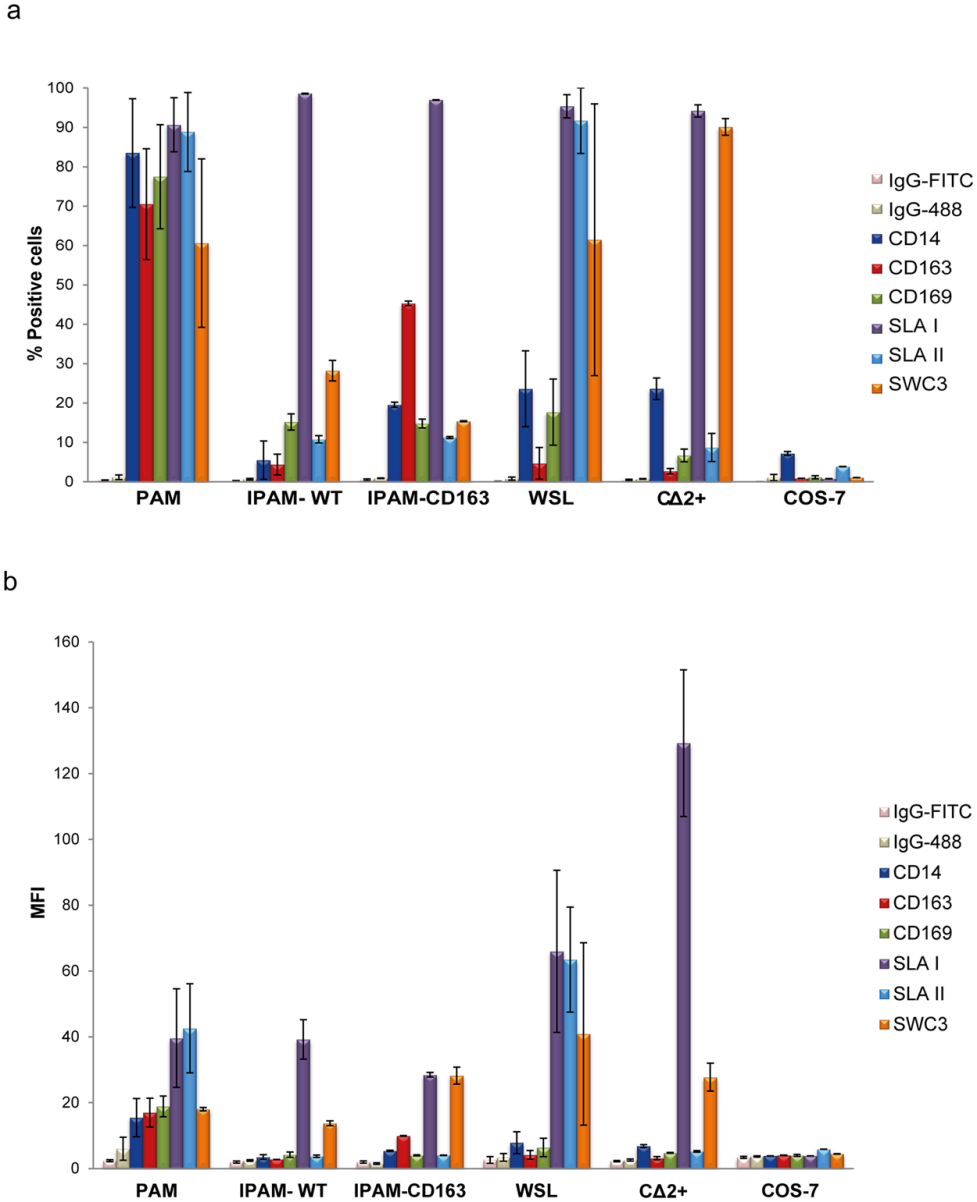
Analysis of membrane receptors in PAM, IPAM-WT, IPAM-CD163, WSL, CΔ2+ and COS-7 cells. Cells were incubated with different antibodies against CD14, CD163, CD169, SLAI, SLAII and SWC3 membrane receptors and the percentage of positive cells **(a)** or mean fluorescence intensity (MFI) **(b)** was analysed by FACS (n ≥ 4, performed in duplicate; mean ± S.D.). An IgG2_b_-FITC (IgG-FITC) and anti-mouse IgGs Alexa Fluor-488 (IgG-488) were used as negative controls for CD14 expression and the other receptors respectively.

**Figure 2:**
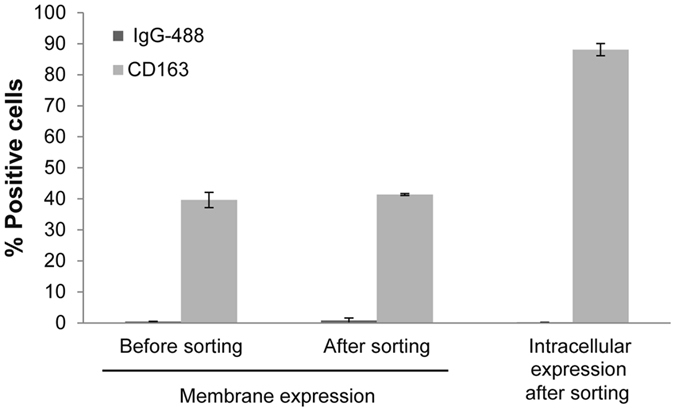
Analysis of membrane and intracellular expression of CD163 on IPAM-CD163 cells. Cells were permeabilized (intracellular expression) or non-permeabilized (membrane expression) before and after sorting and incubated with mouse-anti-CD163 antibody following with an anti-mouse IgGs AlexaFluor-488. Cells were incubated with anti-mouse IgGs AlexaFluor-488 (IgG-488) as background of the cell line. The percentage of positive cells was analyzed by FACS (n = 2; mean ± S.D).

As expected, a high percentage of PAM cells were positive for monocyte/macrophage markers whereas the rest of the cell lines examined had much lower percentages (Fig. 1a and Supplementary Fig. S1). CD14 was found on around 80% of PAM, whereas it was found on only 23% of WSL and CΔ2+, 5.5% of IPAM-WT and 19.5% of IPAM-CD163. Nevertheless, when the expression of SWC3 was examined, we obtained 60% of PAM and WSL, 28% of IPAM-WT, 15% of IPAM-CD163 and 90% of CΔ2+ positive cells. These results indicate that unlike PAMs, which displayed a high percentage of CD14 and SWC3 expression, differences in the percentage of cells expressing CD14 and/or SWC3 markers were found on most of the cell lines analysed. About 70% and 77% of PAM expressed CD163 and CD169 receptors, respectively, with a lower percentage of positive cells found in the rest of cell lines: 5.9% in WSL, 4.4% in IPAM-WT, 45% in IPAM-CD163 and 2.7% in CΔ2+ for CD163 receptor; 16% in WSL, 15.2% in IPAM-WT, 14.8% in IPAM-CD163 and 6.6% in CΔ2+ for CD169. Regarding SLA-II expression, only WSL showed similar levels to those found in PAM. All the porcine cell lines tested expressed the SLA-I porcine marker, and, as expected, none of the analysed receptors were found to be expressed on the monkey epithelial cell line COS-7. When we examined the mean fluorescence intensity (MFI) for these receptors, PAM showed a higher level for CD163 and CD169 compared to the other porcine cell lines; the MFI of the SWC3 in the cell lines was high in comparison with the rest of the receptors analysed, and interestingly, the MFI of SLA-II was slightly higher in WSL that in PAM (Fig. 1b). Taken together, the results indicate that since the MFI of the SWC3 is higher in respect to CD14, WSL, IPAM-WT and CΔ2+ would derive from myeloid lineage, probably representing immature precursors of macrophages, as they are mostly negative for both macrophage maturation markers CD163 and CD169. Regarding IPAM-CD163 cells, they exhibited a more differentiated phenotype, closer to primary PAM cells. However, in our hands, they were still only moderately positive for CD163 (45%). In an attempt to reach a homogenous culture of IPAM-expressing CD163 cells, the membrane CD163 positive cells were sorted by FACS. However, the surface expression of the receptor was lost over the time in culture (Fig. 2). To determine whether this was due to a defect in translocation of CD163 receptor to the cell membrane, or to a down regulation of the synthesis of the protein, we analysed the expression of the receptor in permeabilized and non-permeabilized sorting cells. We detect CD163 in 90% of cells when the intracellular expression was analysed, compared with 40% on the cellular surface, suggesting that some kind of signalling pathway should be interfered in this pool of cells. On the basis of these results, IPAM-CD163 cells were further discarded, as no advantages of IPAM-CD163 vs IPAM-WT were found.

### Analysis of viral protein expression in ASFV-infected porcine cell lines

**Figure 3:**
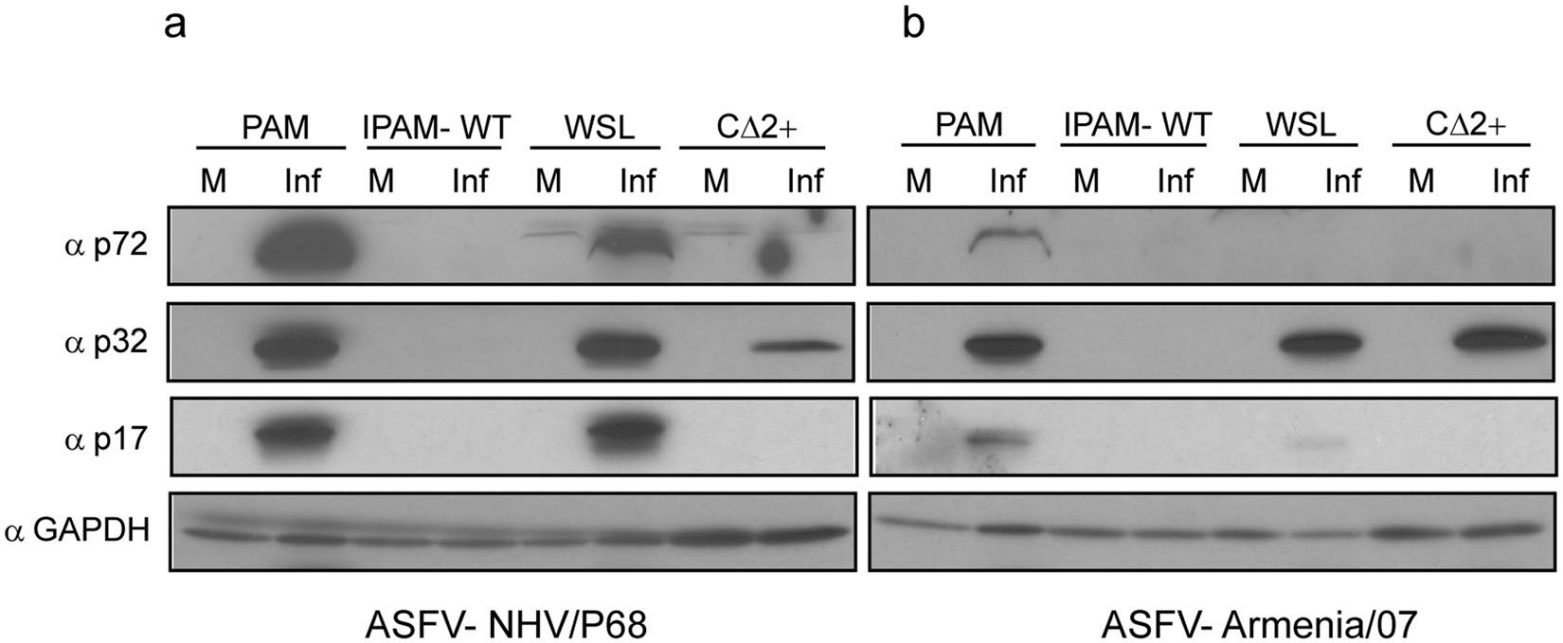
Analysis of viral protein expression in different porcine cell lines. Cells were infected with NHV/P68 **(a)** or Armenia/07 **(b)** isolate (MOI = 3) and at 18 hpi, infected cells were processed and prepared for western blot analysis. Membranes were incubated with antibodies against p32, p72 and p17 proteins; GAPDH was detected as a load control. Gels are representative of two independent experiments. M (mock); Inf (infected).

In order to investigate the susceptibility of IPAM-WT, WSL and CΔ2+ cells to ASFV infection, cells were incubated with both attenuated (NHV/P68) and virulent (Armenia/07) isolates (MOI = 3) and after 18 hpi the expression of several early and late viral proteins was analysed by western blot; PAM were infected as a positive control for ASFV infection. As Fig. 3a shows, the early viral protein p32^53^ was detected at comparable levels in PAM, WSL and CΔ2+ after infection with the attenuated isolate NHV/P68. However, the late proteins p72 and p17^54, 55^ were not detected either in IPAM-WT or CΔ2+, whereas they were clearly expressed during NHV/P68 infection in both PAM and WSL. On the other hand, when cells were infected with the virulent isolate Armenia/07, the pattern of p32 expression was similar to that found during NHV/P68 infection in PAM, WSL and CΔ2+, but, surprisingly, p72 and p17 were only clearly detected in PAM (Fig. 3b). This result reveals either that the infection kinetics of the virulent strain is slower in WSL, or alternatively, that WSL suffer a block at a late step of the Armenia/07 viral cycle.

**Figure 4:**
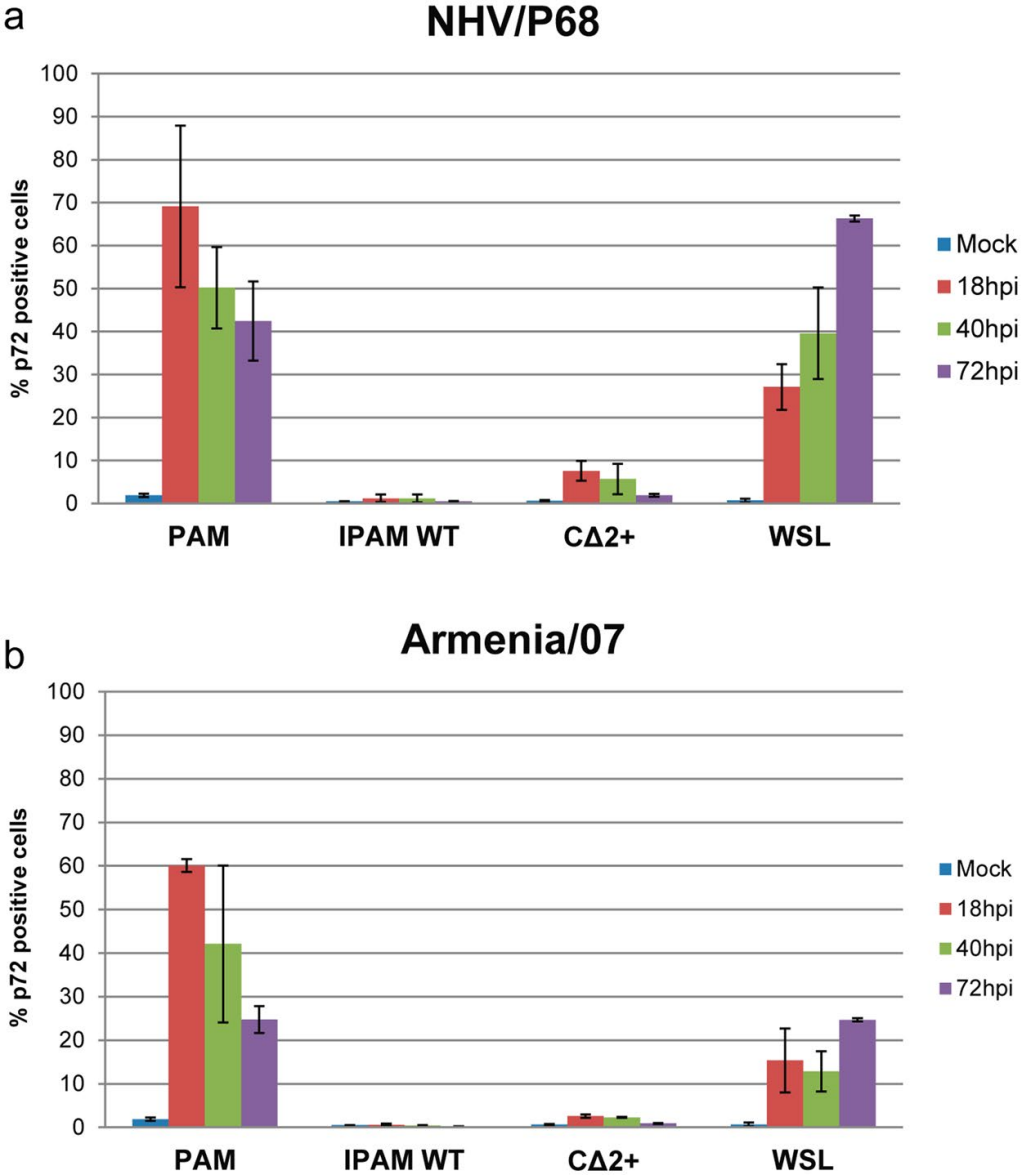
Analysis of PAM, IPAM-WT, CΔ2+ and WSL infected cells. Cells were infected with NHV/P68 (**a**) or Armenia/07 (**b**) ASFV isolates (MOI = 1) and at 18, 40 and 72 hpi, cells were processed for FACS analysis. The percentage of infected cells was detected with a specific Ab against the viral protein p72 (17LD3) following of an anti-mouse IgGs Alexa Fluor-488 (n ≥ 2, performed in duplicate; mean ± S.D.).

In order to further confirm our data and to clarify the different patterns of viral protein expression that occur during NHV/P68 and Armenia/07 infections in WSL, IPAM-WT and CΔ2+, we quantified the number of infected cells by FACS detection of p72, using the specific monoclonal antibody 17LD3 (Ingenasa)^55^. Cells were infected at MOI = 1 either with NHV/P68 (Fig. 4a) or Armenia/07 (Fig. 4b) for 18, 40 and 72 h. The percentage of NHV/P68- and Armenia/07-positive cells was much higher in PAM than CΔ2+ and IPAM-WT at every time-point analysed after infection. In order to improve ASFV infection in IPAM-WT, cells were culture with pig serum for 18 h attempting to increase the expression of CD14 receptor, as previously published^48^. Stimulation of IPAM-WT cells with pig serum neither increased the expression of the receptor nor the level of viral infection after 40 hpi with NHV/P68 strain (Supplementary Fig. S2). Regarding ASFV infection in WSL vs PAM, it is interesting to note that the percentage of p72-positive cells decreased in PAM as infection progressed (from more than 75% ASFV-infected cells at 18 hpi, to about 40% at 72 hpi), whereas the percentage of infected cells increased throughout ASFV infection in WSL cells, reaching a maximum of 65% infected cells after 72 hpi (Fig. 4a). In connection with this, and corroborating the data previously obtained by western blot, much lower levels of p72-positive cells were found when Armenia/07 was used to infect WSL cells. Only around 20% of WSL were infected at 18 hpi, a percentage very similar to that obtained at 72 hpi in PAM. Interestingly, the profile obtained in Armenia/07-infected PAM was similar to that obtained with the attenuated virus although the percentage of p72-positive cells was slightly lower after Armenia/07 infection at the times analysed (Fig. 4b).

**Figure 5:**
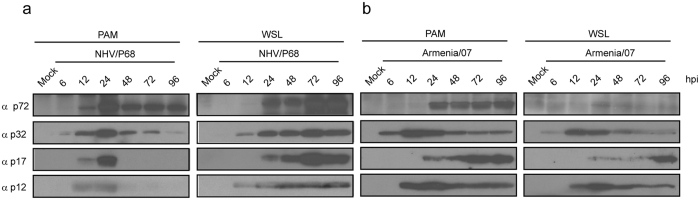
Analysis of viral protein expression in PAM and WSL at late times post-infection. Cells were infected with NHV/P68 (**a**) and Armenia/07 (**b**) isolates (MOI = 2) and viral protein expression was analyzed at 6, 12, 24, 48, 72 and 96 hpi with antibodies against p72, p32, p17 and p12 proteins. Gels are representative of two independent experiments.

To verify the above results, viral protein expression was analysed at 6, 12, 24, 48, 72 and 96 hpi after NHV/P68 and Armenia/07 infection by western blot, using specific antibodies against ASFV-infected cells, previously generated in our lab. During NHV/P68 infection of PAM, the expression of viral proteins such as the early protein p32 and late proteins p72, p17 and p12 increased from 6 hpi (for p32) to 24 hpi, and p32 and p72 expression decreased at 48 hpi and no expression of p17 and p12 was detected (Fig. 5a, left panel). On the other hand, viral protein expression was detected through 96 hpi in NHV/P68 infection of WSL cells (Fig. 5a, right panel), in line with the observed progression of NHV/P68 infection in these cells (Fig. 4a). In contrast, the pattern of late protein expression after Armenia/07 infection of PAM (Fig. 5b, left panel), was apparent at times later than 24 hpi, indicating significant differences between infection of attenuated and virulent strains in macrophages. Interestingly, the pattern of ASFV protein expression in Armenia/07-infected WSL (Fig. 5b, right panel), was different than that found after NHV/P68 infection, as the level of the early protein p32 was not maintained through 96 hpi, and the late protein p17 was not detected until 96 hpi; furthermore, the p72 protein was weakly detected at every time analysed. Taken together, the results indicate that infection of WSL with attenuated virus NHV/P68 progresses satisfactorily, whereas the virulent Armenia/07 strain barely induces synthesis of several of the most important viral proteins in WSL cells.

### Analysis of virus production in ASFV-infected porcine cell lines

**Figure 6:**
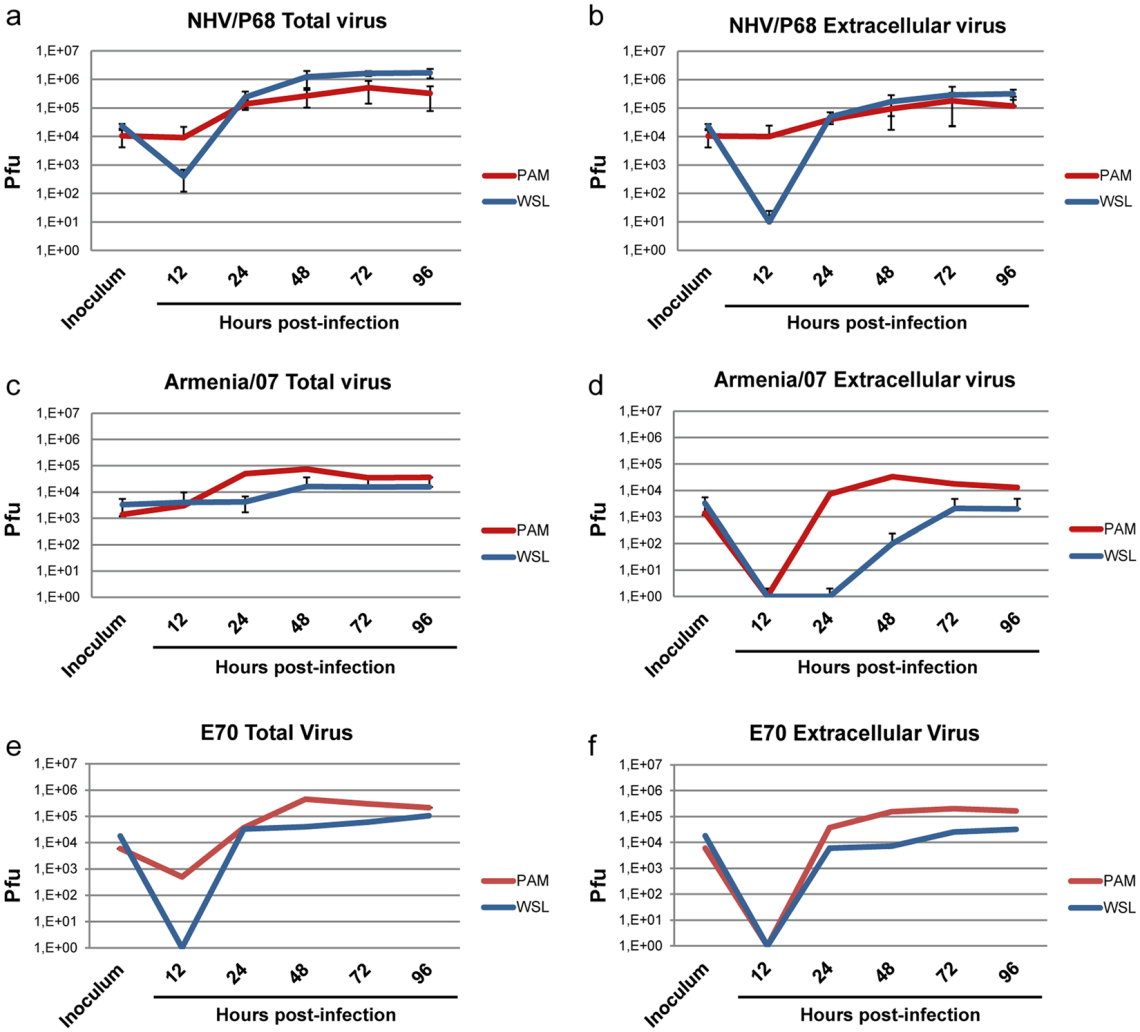
Analysis of ASFV production in PAM and WSL. Cells were infected with NHV/P68 (**a**,**b**), Armenia/07 (**c**,**d**) and E70 (**e**,**f**) isolates (MOI = 0.2) and at indicated times post-infection, total virus (**a**,**c**,**e**) and extracellular virus (**b**,**d**,**f**) was recovered and titrated. The viral production is represented as plaque formation units (Pfu) (n ≥ 2; mean ± S.D.). y-axis is shown on a logarithmic scale.

Virus production was determined to fully assess whether or not the cells are able to sustain production of virulent and attenuated ASFV strains. Neither IPAM-WT nor CΔ2+ were able to produce NHV/P68 ASFV at any of the conditions assayed (Supplementary Fig. S3) in agreement with the lack of ASFV proteins detected by western blot and the lack of p72 detected by FACS. In contrast, WSL was able to produce ASFV. To fully demonstrate this point, and in order to assess the potential use of WSL as a source of infectious ASF virions and LAVs, we compared the viral production of NHV/P68, E70 and Armenia/07 after infection of PAM or WSL. Cells were infected at MOI = 0.2 and both total and extracellular viruses were recovered and titrated by plaque formation in COS-7 cells. The kinetics of total virus production of NHV/P68 in PAM showed that, as expected, virus production increased from 24 hpi, reaching a maximum at 72 hpi, at which viral production began to decrease until 96 hpi. Interestingly, when WSL were analysed for total ASFV production, the virus accumulated faster than in PAM, with twofold more (2.3 × 10^5^ vs 1.3 × 10^5^) at 24 hpi, and almost tenfold more at 48 hpi (1.2 × 10^6^ vs 2.6 × 10^5^). Nevertheless, at 72 hpi the amount of total virus produced by both WSL and PAM tended to be similar (1.6 × 10^6^ vs 8.5 × 10^5^). Importantly, virus production in WSL remained stable whereas PAM production dropped, reflecting the quiescent state of PAM (Fig. 6a). When the extracellular virus was analysed, we found that the amount of shed virus is very similar between PAM and WSL, with only a twofold difference in WSL at 96 hpi (Fig. 6b). These results indicate that WSL perfectly sustain NHV/P68 infection and, since they are dividing, the viral titer increased over time, probably due to several cycles of productive infection. We next investigated the production of Armenia/07 in PAM and WSL, with good production of both total and extracellular virus at 48 hpi in PAM (though lower than NHV/P68). Most importantly, the total and extracellular production of Armenia/07 in WSL was lower (Fig. 6c and d). Regarding total virus, after 24 hpi viral production in WSL was tenfold less than in PAM (5 × 10^4^ vs 4,2 × 10^3^), increasing by half a logarithmic unit from 24 to 48 hpi and maintained at this level through 96 hpi. Extracellular Armenia/07 particles were detected after 48 hpi, although the titer was lower than the initial inoculum. Progeny of Armenia/07 WSL infections were also titered in PAM by hemadsorption obtaining same results (Supplementary Fig. S4). Furthermore, when we reproduced these experiments using the ASFV strain E70, a virulent genotype I ASFV strain, the viral production was similar to Armenia/07 (Fig. 6e and f), suggesting that the relatively low viral production in WSL is associated with virulence, independent of viral genotype. Finally, we found that after several passages of NHV/P68 strain in WSL as well as in PAM or COS-7 cells, the stability of the viral progeny was maintained, indicating that along passages in WSL, the virus does not lose its replicative capacity *in vitro* (Supplementary Fig. S5). Similar results were obtained after either five or ten passages of ASFV in WSL, by analyzing the infection in PAM by FACS with a specific antibody against viral p72 as showed in Supplementary Fig. S6.

Moreover, in order to determine if the virus obtained after several passages in WSL is still able to infect pigs, animals were inoculated with NHV/P68 isolate and clinical score, viremia, antibody titers and survival rate were analysed. As a results shown, pigs developed variable clinical signs starting at 14 dpi, more intense in the animals immunized with the high dose (10^7^ TCID50/ml) of the ASFV (Supplementary Fig. S7). The predominant lesions, previously described in chronic type ASFV infection^56–58^, included slightly raised body temperatures, necrotic skin areas and joint swelling over the period of a month, cyanosis in ears and gradual weight loss. One inoculated pig (AT3) was slaughtered at 27 dpi due to the severe chronic type lesions. The ASFV genome was detected by PCR in all tissue samples obtained from this animal. After three passages in PBM cells the ASFV was recovered from 9 (50%) out of the 18 positive PCR tissues (Supplementary Table S1). The remaining domestic pigs were slaughtered at 52 (AT4, AT1), and 72 (AT2) dpi (Supplementary Fig. S7). Nonspecific internal gross lesions were found at the necropsy, although high numbers of viral genome copies were detected in skin lesions from the joints collected from the animals AT2 (72dpi) and AT4 (d52pi). Viremia was detected to a variable extent in the inoculated pigs (Supplementary Fig. S8). In pig AT3, which developed a more intense chronic form of infection, viremia was detected at 7 dpi and remained positive until slaughtered at 27 dpi. In the animals with mild clinical signs, the first PCR positive result was identified in the pig AT1 at 14 dpi, and at 28 dpi in the AT4 coincident with the appearance of the clinical signs. The viremia lasted for approximately over one month although showing intermittent and weak peaks of viremia (Ct > 35) from 43 to 52 dpi. No viremia was detected in the pig AT2, which developed a subclinical type of infection. Regarding the presence of ASFV-specific antibodies, seroconversion was detected in all pigs using the IPT at 7 (AT3, AT4) and 14 (AT1, AT2) dpi, yielded very high values (>10^5^) during all the observation period (Supplementary Fig. S8). Therefore, these data shows the ability of the NHV/P68 ASFV strain produced in WSL cells to infect domestic pigs, inducing a similar infection pattern to that induce when animals are inoculated with the NHV/P68 ASFV strain obtained in PAM.

### Viral factory formation in WSL

**Figure 7:**
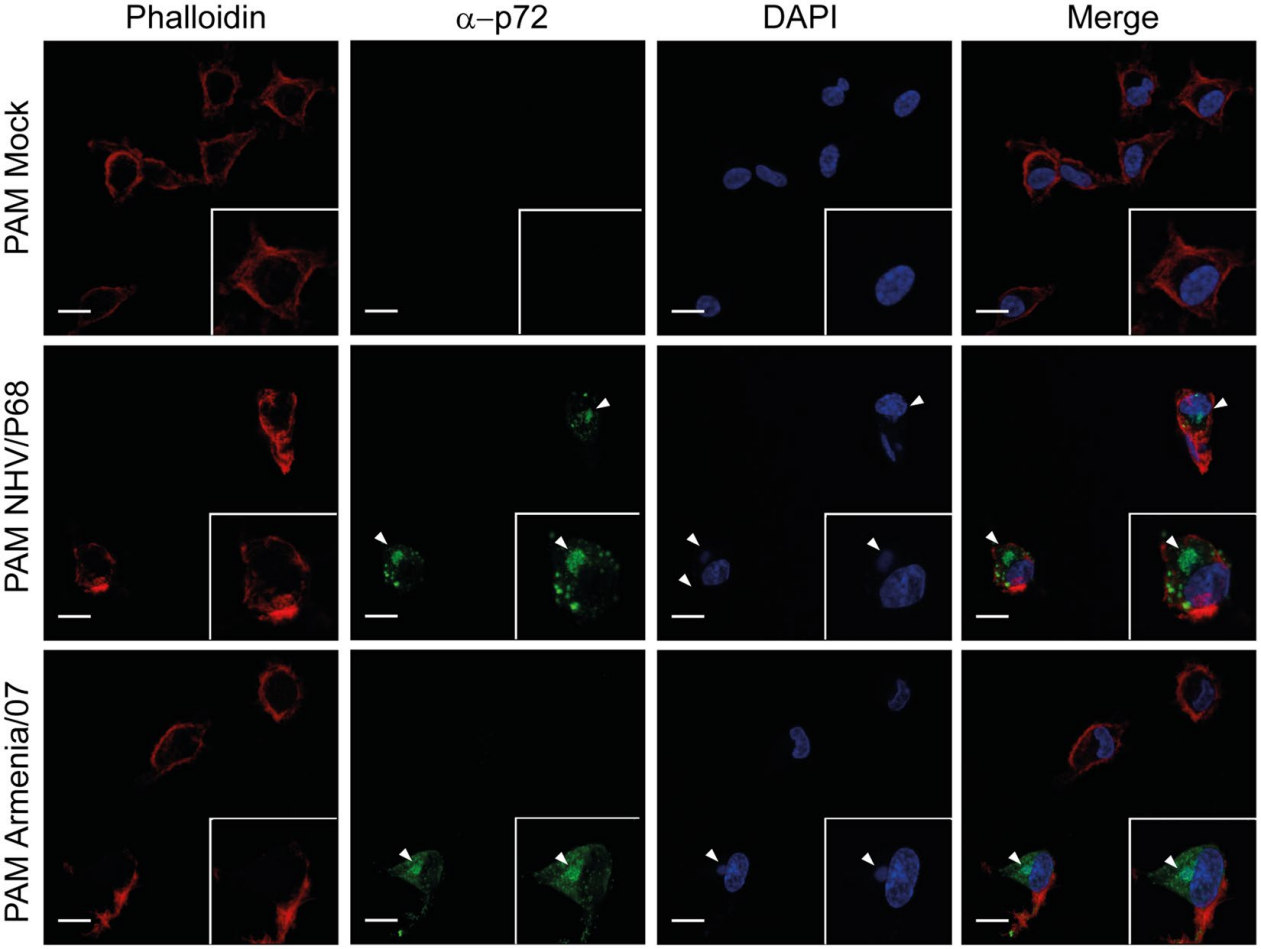
Viral factory pattern in PAM. Cells were infected with NHV/P68 and Armenia/07 strains (MOI = 1), fixed at 16hpi and incubated with phalloidin-TRITC, anti-p72 antibody and DAPI to respectively stain actin filaments, viral particles and cellular and viral DNA. Z-slides images were taken by CLSM and represented as a maximum of z-projection. Arrowheads show the viral factories visualized by anti-p72 MAb and with DAPI to stain the viral DNA. Images are representative of two independent experiments.

**Figure 8:**
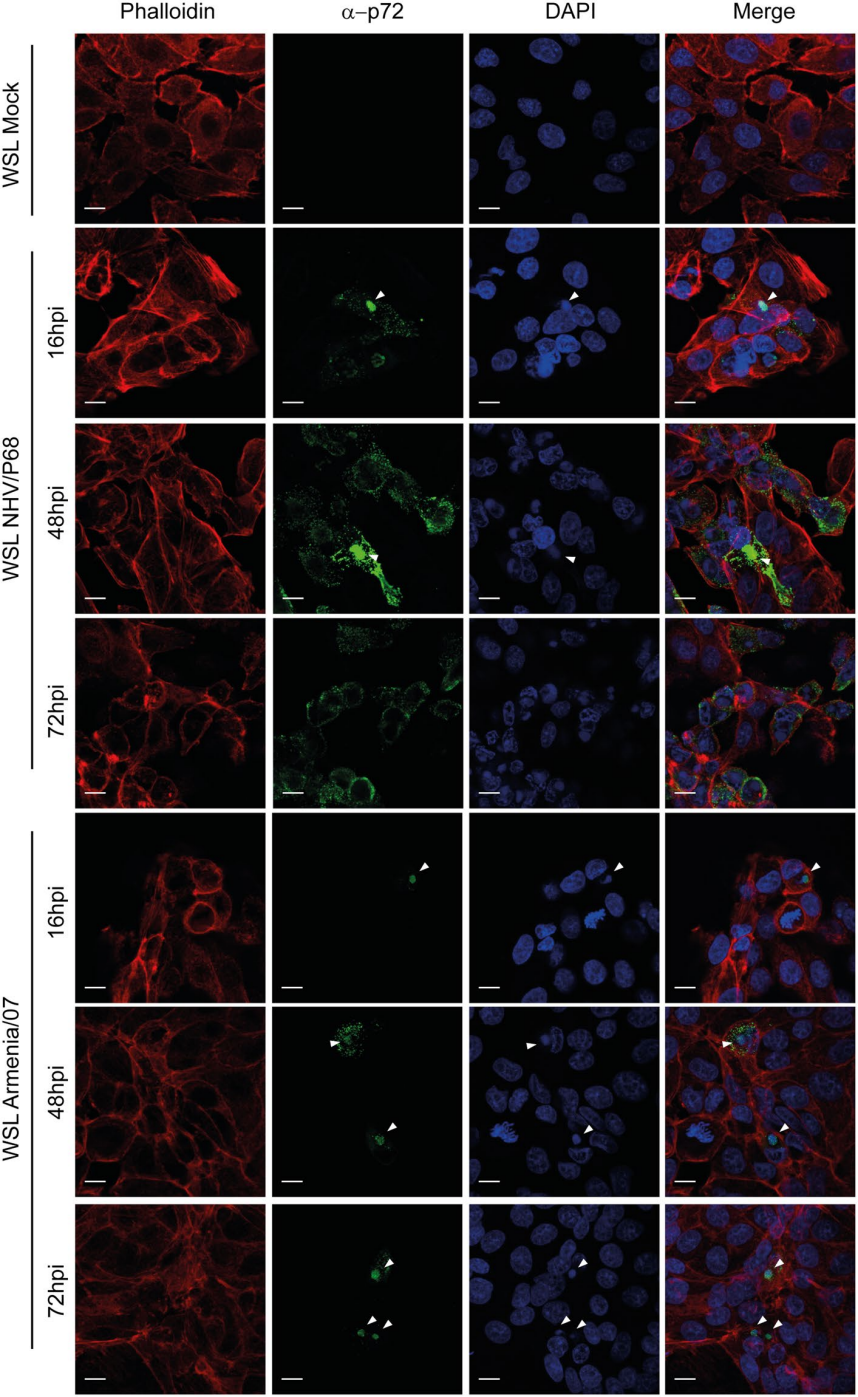
Viral factory pattern in WSL. Cells were infected with NHV/P68 and Armenia/07 strains (MOI = 1), fixed at indicated times and incubated with phalloidin-TRITC, anti-p72 antibody and DAPI to stain respectively actin filaments, viral particles and cellular and viral DNA. Z-slides images were taken by CLSM and represented as a maximum of z-projection. Arrowheads show the viral factories visualized by anti-p72 MAb and with DAPI to stain the viral DNA. Images are representative of two independent experiments.

Since the above results indicate lower production of virulent ASFV in WSL than the attenuated NHV/P68 strain, we hypothesized a defect may occur in the formation of virulent viral factories. To test this, WSL and PAM were infected with both attenuated and virulent isolates at MOI = 1 and subsequently processed for immunostaining and CLSM analysis. The viral factories were visualized by anti-p72 (17LD3) MAb and with DAPI to stain the viral DNA. Whereas PAM displayed a typical factory distribution with both ASFV isolates (Fig. 7), in WSL infected with NHV/P68, factories were present in some cells, but with abundant punctated p72, suggesting virus particles present all over the cells (Fig. 8). On the other hand, the number of p72-positive WSL was much lower in Armenia/07 at every time-point analysed, indicating that infection with the virulent isolate is not as efficient (consistent with previous results). Armenia/07 rendered typical viral factories in terms of shape, perinuclear localization and p72 presence. Importantly, the number of infected cells did not further increase over time in comparison with NHV/P68 strain, suggesting that some kind of block at a step of the infection may be occurring (Fig. 8).

## Discussion

Primary culture systems for virus infection offer advantages for biological and immunological *in vitro* studies since it mimics *in vivo* behaviour. However, experimentation with primary cells is expensive and time-consuming as well as highly variable due to the cell donor and the batch to batch variation. ASFV shows a strict tropism for cells from the monocyte/macrophage lineage^1, 17^ and a suitable porcine cell line for *in vitro* studies has not yet been established. This complicates not only the setup of *in vitro* host-related assays, but also the development of a vaccine against ASFV and the production of non-adapted isolates for laboratory research. In the present study, productive infection of the attenuated NHV/P68 and virulent Armenia/07 and E70 strains was investigated in three porcine cell lines – IPAM-WT^48^, WSL^50^ and CΔ2+^52^ - concluding that only WSL are able to support production of attenuated NHV/P68 ASFV strain, whereas the virulent strains were not produced efficiently in any of the cells.

The presence of specific receptors on the host cell membrane is a determinant for viral tropism and internalization. It has been reported that monocytes/macrophages with a higher maturation and differentiation state are the cell set preferentially infected by ASFV^24, 43^. In this respect, whereas CD14 and SWC3 are considered as myeloid markers, CD163 and CD169 are related to the maturation and differentiation of the lineage^25, 26, 30^, and it has been published that the higher percentages of mature monocytes/macrophages infected with the virulent ASFV isolate E75 correlated with a higher expression of CD163^43^. Moreover, magnetically sorted CD163-positive blood monocytes were more susceptible to infection than monocytes lacking CD163, and the use of specific antibodies blocking the receptor resulted in different degrees of ASFV inhibition, suggesting that CD163 may be involved in the first steps of ASFV infection^43^. On the other hand, another study stated that CD163 is not needed for ASFV infection^44^ and moreover, pigs lacking CD163 have been shown to be infected by Georgia 2007/1 ASFV^45^. This suggests that this receptor, although possibly involved in early steps *in vitro*, may not affect ASFV infection *in vivo*. In the present work, a set of receptors related to the monocyte/macrophage lineage on IPAM-WT, IPAM-CD163, WSL and CΔ2+ cells was determined in order to assess whether or not these cells displayed a mielomonocytic phenotype. In each case, only a low percentage of cells expressing CD14, CD163 and CD169 were found. In this regard, our data for CΔ2+ was consistent with previous reports^52^. For IPAM-WT, there was a lower percentage of CD14 positive cells, similar to those obtained in COS-7 cells, even though around 25% of SWC3 positive cells showing a high MFI were found, accordingly to previously published^59^. Regarding WSL, only around 23% expressed at high levels the CD14 marker, but interestingly, the expression of SWC3 was found to be still higher. Cells showed a remarkable variability that suggests that even when some cells are precursors from myeloid linage, the culture is not homogeneous and contains cells in other steps of maturation. We also obtained a low percentage of CD163- and CD169-positive WSL cells, with a lower MFI than in PAM. However, the number of WSL expressing SLA-II was similar suggesting that both cell types could be able to present viral antigens in the context of this SLA class. Nevertheless, the MFI for SLA-II was clearly higher in WSL than in PAM, indicating an important role for this receptor in these cell lines. The fact of the expression profile of the different myeloid receptors (CD14 and SWC3) is somewhat different, could reflect slight differences in the staining patterns of the Abs used. The low percentage of cells expressing CD169 and CD163 indicates that although some cells express myeloid markers, they are not mature monocytes or macrophages. Our data pointed out that there is no direct correlation between CD14 expression and ASFV infection since, as shown in Fig. 1, both the percentage of WSL and CΔ2+ CD14 positive cells as well as MFI are very similar, whereas infection levels in WSL are higher than in CΔ2+ (Fig. 4). The fact that COS-7 cells, which do not express any of the receptors associated with the myeloid lineage, have been widely used to sustain infection of several ASFV isolates without prior adaptation suggests that these receptors are not strictly required for productive infection. However, it cannot be discarded that virus progeny generated in COS-7 don’t display similar viral features as those produced in monocytes/macrophages or WSL. In fact, differences in protection *in vivo* were found when recombinant ASFV viruses produced in COS-7 were used as LAVs (Revilla Y *et al*., manuscript in preparation). This is an important point to take into account when using cell models that are not the natural target host cell for the production of vaccines.

Based on the results of this study, only WSL cells (apart from PAM) are useful to study ASFV infection in terms of protein expression and viral production, and importantly, we demonstrate that the virus keeps its ability to infect and replicate both in porcine macrophages and pigs after several passages in WSL cells. Results obtained with other cell lines such as IPAM-WT showed that neither early nor late viral proteins were detected after infection with attenuated or virulent virus, consistent with the barely detectable percentage of infected cells. These data are in line with a previous work which described that IPAM-WT cells were sensitive to only certain strains^48, 59, 60^. In the case of CΔ2+, this is the first report about its susceptibility to ASFV, and in our hands, only the expression of early viral protein p32 was detected by western blot after NHV/P68 and Armenia/07 infection, in agreement with the inapparent infection of cells as determined by FACS analysis of p72. Our data also showed that IPAM-WT and CΔ2+ cells do not support productive infection, as no viral particles could be found in the infection supernatants from either of them at a MOI used. However they are sensitive to both attenuated and virulent ASFV strains, in terms of viral entry and early viral protein expression.

Interestingly, the data presented here indicate that WSL cells may be a good model for production of attenuated ASFV strains over long periods of infection, although it is necessary to keep in mind that they cannot be considered macrophages based on the presented data. This obviously precludes their use in the study of the molecular mechanism of ASFV-macrophage interaction. However, what we observed is that several rounds of infection take place after infection of WSL, giving the possibility that WSL could be used as a kind of “continuous culture” for ASFV production. This is completely different than what has been observed during infection of macrophages, where viral production drops drastically after 72 hpi, suggesting that the culture was completely infected by that time, as obviously PAM are not able to keep dividing. Surprisingly, WSL cells seem to be less productive for virulent ASFV isolates, including genotype I and genotype II strains, in comparison to PAM. Our results are in line with a report that described that WSL produced higher titers of attenuated than virulent ASFV strains^59^, and that they are useful to produce recombinant NHV/P68 virus^50^. Interestingly, both early and late proteins were detected during infection of WSL with Armenia/07, with the sole exception of p72, which was only barely detected. We cannot discard that other antibodies against p72 may have shown a different pattern of detection, but it is clear that under the same conditions, p72 was detected only during NHV/P68 infection of WSL. Further studies must be done to determine if p72 is not efficiently expressed, or rather undergoes sequence or structural modification during infection by virulent strains. We also speculate that the difference of CD14-positive PAM vs WSL suggests the possibility that some steps of the entry into the host cells by virulent strains may be linked to CD14. In this respect, it is also intriguing that SLA II was highly expressed on WSL cells (even higher than on PAM), as SLA II has been reported to be upregulated during ASFV infection^43^. Overall, our work offers an accurate, comparative and quantitative analysis of the reliability of several porcine cell lines as possible models, both for research on ASFV-host interaction molecular biology and for vaccine development, with special importance for the production of LAVs.

## Materials and Methods

### Cells and Viruses

PAM were obtained by bronchoalveolar lavage as previously described^60^. COS-7 (CLR-1650) from African green monkey kidney, LM-929 cells (CmCL 1.2) from mouse and IPAM WT cells (CRL-2845) from swine lungs were obtained from the American Type Culture Collection (ATCC). IPAM-CD163 were kindly gifted by Dr. Changhee Lee (College of Natural Sciences, Kyungpook National University, South Korea), WSL cells (from wild boar lung) by Dr. Günther Keil (Friedrich-Loeffler-Institut, Federal Research Institute for Animal Health, Germany) and CΔ2+ (porcine monocytes) by Dr. Chitko-McKown (U.S. Meat Animal Research Center, Clay Center, United States). The cell lines IPAM WT^48^, IPAM-CD163^49^, WSL^50, 51^ and Cdelta2+^52^ were obtained as previously described by the authors. PAM were cultured in Dulbecco’s modified Eagle’s medium (DMEM) supplemented with 10% of pig serum (PS) (Sigma) and COS-7 in DMEM with 5% of fetal bovine serum (FBS) (Invitrogen Life Technologies). LM-929 were used as a source of colony-stimulating factor (M-CSF)^61^ for CΔ2+ cells and were cultured in Roswell Park Memorial Institute (RPMI) media supplemented with 5% FBS. Supernatants were stored at −80 °C, and then passed through a 0.45 micron filter prior to use. IPAM WT, IPAM-CD163, WSL and CΔ2+ were grown in RPMI supplemented with 10% of FBS and in the case of CΔ2+, 10% of LM-929 supernatant. When indicated, IPAM WT were cultured with RPMI 10% PS for 18 h. Cells were grown at 37 °C and 7% CO_2_ atmosphere saturated with water vapour in a culture medium supplemented with 2 mM L- glutamine, 100 U/ml gentamicin and 0,4 mM of nonessential amino acids. The field attenuated ASFV strain NHV/P68 (non hemoabsorbing virus Portugal 68)^58^ and the field virulent strains Armenia/07^4^ and E70 were propagated and obtained in PAM. Briefly, PAM were infected with ASFV at a multiplicity of infection (MOI) of 0.5 in DMEM with 10% PS, and at 96 hpi or after total cytopathic effect was reached, cells were harvested and stored at −80 °C. NHV/P68 isolate obtained in PAM were passaged in WSL cells before use it to infect PAM (five or ten times) or pigs (ten times passaged). For *in vivo* experiments viral titers were estimated using Reed and Muench’s method and expressed as 50% haemadsorbing doses per ml (HAD50/ml) per sample.

### Virus infection

Cells were mock-infected or infected with NHV/P68, Armenia/07 or E70 ASFV strains at different MOI ranging from 0.2–3 pfu/cell in media supplemented with serum according to cell type. After 90 min at 37 °C (adsorption period) the virus was removed, cells were washed twice with medium and incubated at 37 °C until the indicated times.

### Fluorescence Activated Cell Sorting (FACS)

The analysis of both membrane receptors and the intracytoplasmic expression of the viral protein p72 were performed by FACS. For membrane receptor analysis, after two days in culture, 1 × 10^6^ cells were detached with 2 mM EDTA for 5 min at 37 °C. In order to block Fc receptors, cells were incubated with PBS with 5% PS for 15 min on ice and washed once with PBS-Staining Buffer (PBS with 0.01% sodium azide, 0.5% BSA). Then, cells were incubated for 30 min at 4 °C with 50 µl of antibodies against CD163 (2A10), CD169 (1F1/CR4), SLAII (1F12) and SLAI (4B7/8), all of which were kindly gifted by Dr. Javier Dominguez (INIA, Spain). Cells were washed and incubated in darkness at the same conditions with a donkey anti-mouse IgGs Alexa Fluor-488 (1:500) or 1 µl of CD14-FITC (MIL-2; Bio-rad MCA1218F) conjugated antibodies. For intracellular staining of p72, at the indicated times post-infection, cells were detached with 2 mM EDTA and fixed with 2% paraformaldehyde for 30 min at 37 °C. The Fc receptors were blocked and then cells were permeabilized with PBS-Staining buffer with 0.2% saponin for 15 min at room temperature (RT). Detection of infected cells was performed by incubation with an anti-p72 monoclonal antibody (17LD3; Ingenasa) (1:100) for 30 min at 4 °C, followed by incubation with a donkey anti-mouse IgGs Alexa Fluor-488 (diluted 1:500) or an donkey anti-mouse IgGs Alexa Fluor-647 (1:500) (Thermo Fisher) in the same conditions. Finally, 2 × 10^4^ cells were analysed in a FACSCalibur flow cytometer (BD Science) to determine the percentage and mean fluorescent intensity (MFI) of infected cells. Controls used to determine the background of each cell line were, respectively, a mouse irrelevant IgG2_b_-FITC (BD Bioscience)for expression of CD14 and donkey anti-mouse IgGs Alexa Fluor-488 for the expression of the other receptors. For the analysis of the infection, the negative background was determined with the mock sample stained with the antibody anti-p72 plus Alexa Fluor-488 or Alexa Fluor-647. All FACS analyses were performed at least in triplicate and displayed as the average percentage of positive cells and MFI. For sorting of IPAM-CD163 cells, 1 × 10^7^ cells were incubated with 0.5 ml of CD163 antibody (2A10) followed by IgGs Alexa Fluor-488 (1:500) at the same conditions described above and cells expressing CD163 on cellular membrane were sorted by FACSVantage. A table of antibodies used is shown in Supplementary information online (Supplementary Table S2).

### Electrophoresis and Western Blot

At indicated times post-infection cells were washed with PBS and lysed in RIPA buffer (50 mM Tris-HCl pH 7.4, 150 mM NaCl, 1% Triton, 0.5% Deoxycholate, SDS 0.1%) supplemented with protease and phosphatase inhibitor cocktail tablets (Roche). Protein concentration was determined by a Pierce BCA Protein Assay kit (Thermo Scientific). Cell lysates were fractionated by SDS-PAGE and electrophoretically transferred to an Immobilon extra membrane (Amersham) and the separated proteins reacted with specific primary antibodies. The following antibodies were used: polyclonal anti-p72 (dilution 1:2000), anti-p32 (dilution 1:1000), anti-p12 (dilution 1:4000); anti-p17 (dilution 1:500); anti-GAPDH (dilution 1:10.000). Membranes were exposed to horseradish peroxidase-conjugated secondary antibodies (dilution 1:5000) followed by chemiluminescence (ECL, Amersham Biosciences) detection.

### Confocal Laser Scanning Microscopy (CLSM)

Cells were grown on glass coverslips and, at indicated times post-infection, were fixed with 4% paraformaldehyde for 20 min and permeabilized with PBS-0.2% Triton X-100 for 15 min at RT. Viral particles and virus factories were stained with an anti-p72 monoclonal antibody (17LD3) (diluted 1:250 in PBS with 5% BSA) for 60 min at RT, followed by incubation with an anti-mouse Alexa Fluor-488. Alexa TRICT-phalloidin and DAPI (dilution 1:500) were used to stain actin filaments and DNA, respectively. Samples were analyzed by CLSM (Zeiss LSM 710/LSM 710 NLO and ConfoCor 3) with a 63x oil immersion objective and Z-slices per image were collected and displayed as maximum z-projection of horizontal slices (*x-y* plane). For presentation of images in the manuscript, LSM images were imported into Image J software for analysis.

### Viral Titration

Extracellular virus and total virus produced by cells were collected at indicated times post-infection and conserved at −80 °C. Titration was performed by plaque assay on subconfluent COS-7 cells by adding 10-fold serial dilutions of the virus samples. After 90 min at 37 °C, wells were overlaid with 50/50 agar-medium solution (agar 1%-DMEM2X medium with 10% FBS). At 5–7 dpi, COS-7 cells were dyed with 2% crystal violet in 5% formaldehyde and lysis plaques formed in the monolayer were counted. Virus production obtained in WSL cells was also titrated by hemadsorption assays in PAM as described^60^.

### *In vivo* experimental procedures

Pigs were infected with NHV/P68 isolate after ten passages in WSL. The procedures and sampling analysis are explained in detail in Supplementary information.

## Electronic supplementary material

Supplementary Information
