# Supplementary material for: Phenotyping and susceptibility of established porcine cells lines to African Swine Fever Virus infection and viral production
Source: Sci Rep. 2017 Sep 4;7:10369. doi: 10.1038/s41598-017-09948-x (PMC5583235; doi:10.1038/s41598-017-09948-x)
Supplement: Supplementary file 1 — Supplementary Information [file 41598_2017_9948_MOESM1_ESM.pdf]

Phenotyping and susceptibility of established porcine cells lines to African Swine Fever Virus infection and viral production

Elena G. Sánchez[1], Elena Riera[1], Marisa Nogal[1], Carmina Gallardo[5], Paloma Fernández[1], Raquel Bello-Morales[3], José Antonio López-Guerrero[3], Carol G. Chitko-McKown[4], Jürgen A. Richt[2] and Yolanda Revilla[1*]

Material and Methods: In vivo experimental procedures

1. **Experimental design and sampling collection.**

In vivo experiment was conducted at the Biosafety level 3 (BSL3) animal facilities at CISA-INIA in accordance with Spanish and European regulations and EC Directive 86/609/EEC, and following the recommendation 2007/526/EC for the accommodation and care of animals used for experimental and other scientific purpose. The animal experiments were approved by the Spanish Ethical and Animal Welfare Committee under reference number PROEX 338/15. The study included four Landrace x Large White domestic pigs with an age of eight to twelve weeks which were divided into two experimental groups placed in separate units. All pigs were intramuscularly (i.m) immunized either with 10^5 50% tissue culture infectious doses per ml (TCID50/ml) of NHV/P68-WSL (pigs AT1, AT2) or with 10^7 TCID50/ml of NHV/P68-WSL. Severity of the disease was expressed by a clinical scoring, obtained by adding the score of eight clinical signs, recorded daily as previously described [56]. Paired EDTA-blood and serum samples were collected from pigs at 7, 14, 21, 28, 35, 43, 48, 51, 55, 58, 62, 65 and 72 day post-immunization (dpi). Negative control samples were collected at day 0, the day of inoculation. Twenty different types of tissues and organs were obtained from each necropsied animal including; liver, spleen, tonsil, heart, lung, kidney, submandibular, retropharyngeal, inguinal, popliteal, mesenteric, mediastinal, gastro-hepatic, splenic and renal lymph nodes, bone marrow and intra-articular tissues of joints.

2. **Sample analysis**

ASF virus detection: DNA was extracted from all organ homogenates and blood samples using the High Pure PCR Template Preparation kit (Roche Diagnostics GmbH, Roche Applied Science, Mannheim, Germany). Briefly, 10% (w/v) clarified homogenized tissue suspensions were prepared in phosphate-buffered saline using field and experimental tissue samples. For amplification of the ASFV genomic DNA the Universal Probe Library (UPL) real-time PCR was carried out using undiluted extracted DNA for each sample. Samples with recorded threshold cycle number (Ct) < 40.0 were considered positive and samples with no recorded Ct value were considered negative. Virus isolation and titration were performed using porcine peripheral blood macrophages (PBM) (Carrascosa et al., 2011).

Antibody detection: The ASFV antibody titres were determined in serum samples by end-point dilution using the indirect immunoperoxidase test (IPT) as described by the EURL (2014).

Supplementary Figures:

Supplementary Figure S1: Analysis of membrane receptors in PAM, IPAM-WT, IPAM-CD163, WSL, CΔ2+ and COS-7 cells. Cells were incubated with different antibodies against CD14 **(a)**, CD163, CD169, SLAI, SLAII and SWC3 **(b)** membrane receptors. The histograms represent the fluorescence distribution of the analyzed populations of one representative experiment (arbitrary units) (n ≥ 4, performed in duplicate; mean ±S.D.) . $IgG2_b$-FITC (IgG-FITC) and anti-mouse IgGs Alexa Fluor-488 (IgG-488) were used as negative controls.

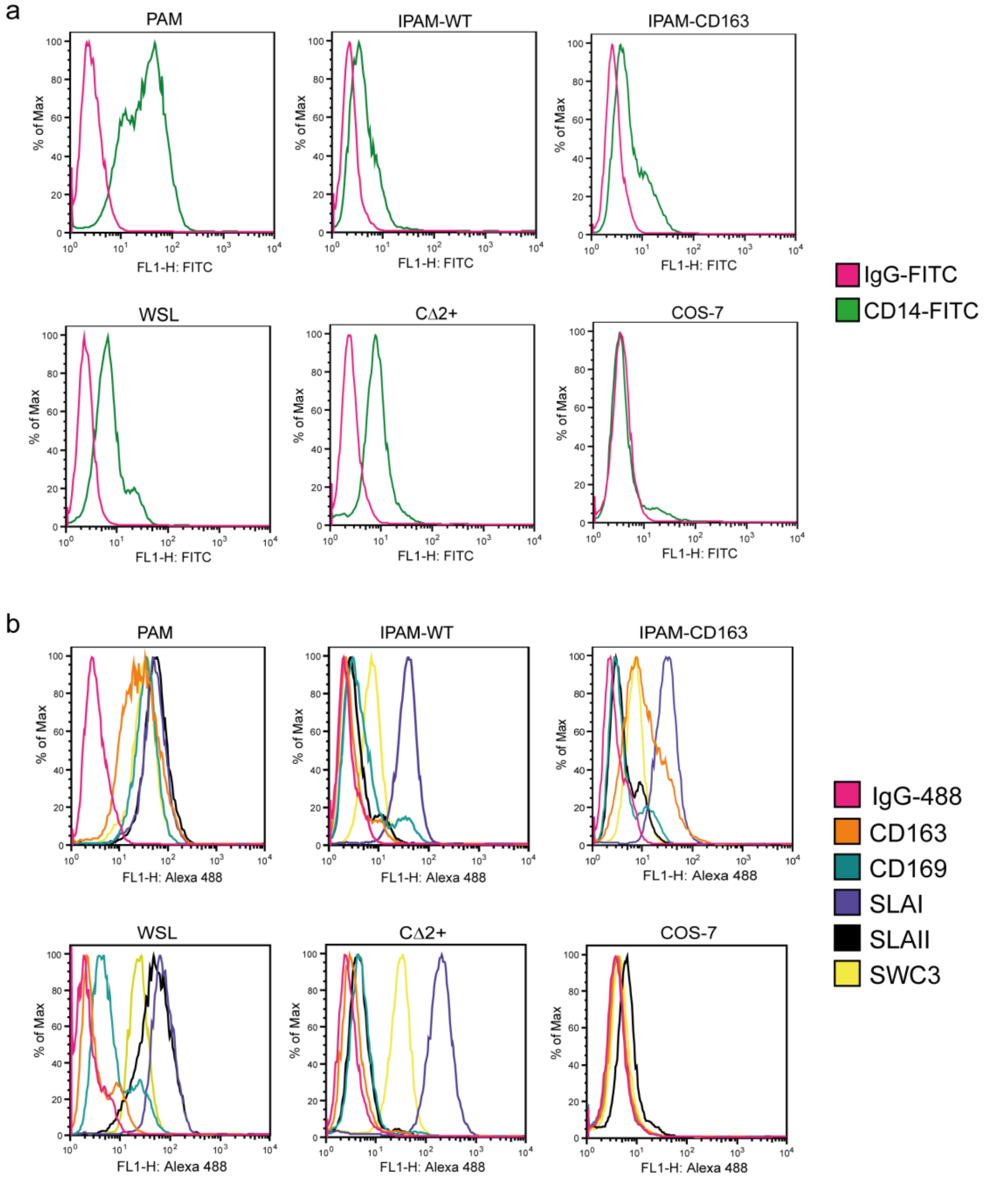

Supplementary Figure S2: CD14 expression and ASFV infection in IPAM-WT stimulated with pig serum: Cells were culture with fetal bovine serum (FBS) or pig serum (PS) for 18 h and then CD14 expression was analysed by FACS with a specific antibody CD14-FITC **(a)**. After this stimulation, cells were infected with NHV/P68 isolate (MOI=1) for 40 h and the percentage of infected cells was analysed by FACS with a specific Ab against the viral protein p72 (17LD3) following of an anti-mouse IgGs Alexa Fluor-647 **(b)**. The graphics represent the percentage of positive cells (n ≥ 1; mean ±S.D.). An $IgG2_b$-FITC (IgG-FITC) was used as negative control for CD14 expression.

a

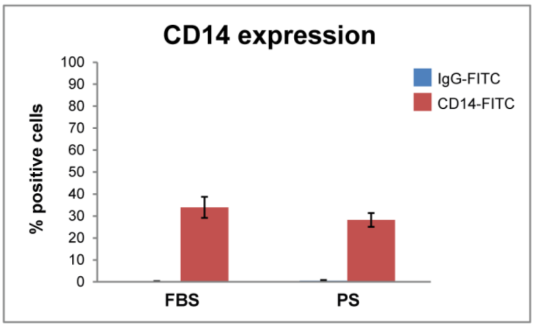

b

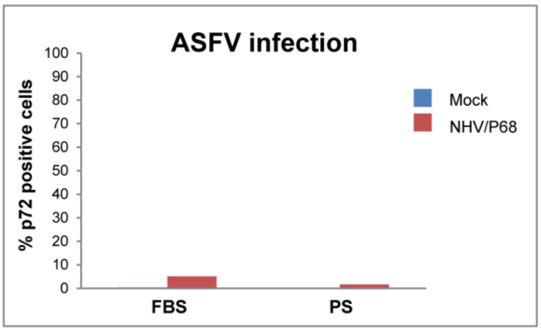

Supplementary Figure S3: ASFV production in IPAM-WT and CΔ2+ cells. Cells were infected with NHV/P68 isolate (MOI 0.2) and at 72 hpi total virus produced was recovered and titrated in COS-7 cells. The viral production is represented as plaque formation units (Pfu). y-axis is shown on a logarithmic scale.

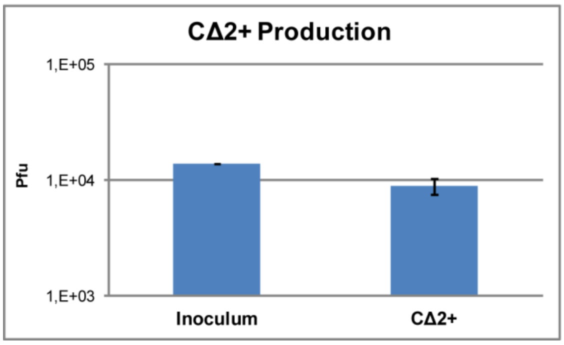

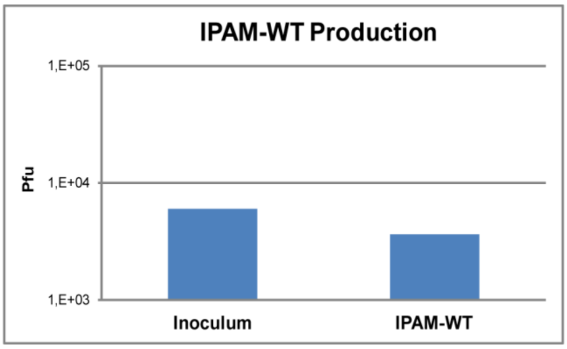

Supplementary Figure S4: Analysis of WSL-Armenia/07 production by hemadsorption assays on swine macrophages. WSL cells were infected with Armenia/07 isolate (MOI= 0.2) and at indicated times of infection, total and extracellular virus were recovered and titrated on swine macrophages by hemadsorption assays. The viral production is represented as hemadsorption units (HADU). y-axis is shown on a logarithmic scale.

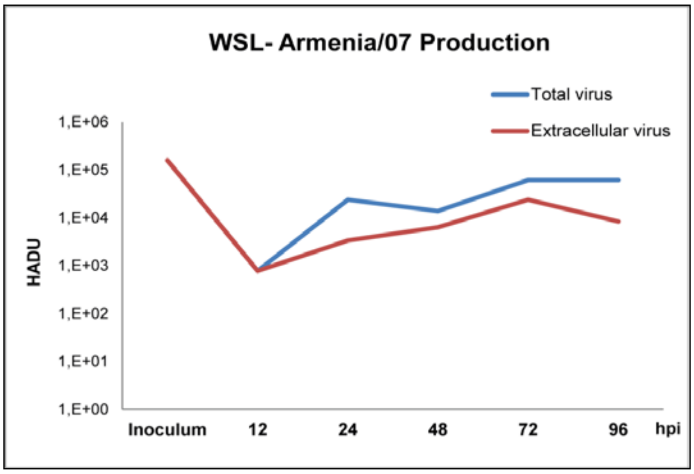

Supplementary Figure S5: Analysis of NHV/P68 viral production along passages in WSL, PAM and COS-7 cells. ASFV NHV/P68 strain was passed four times consecutively by WSL cells (1st, 2nd, 3rd and 4th passage through WSL), followed once by PAM (5th PAM) and by COS-7 cells (6th COS-7). In all cases cells were infected with MOI=1 and after 96 h total virus production was collected and titrated. Viral titre was calculated in each case by plating assays in COS-7 cells. y-axis is shown on a logarithmic scale.

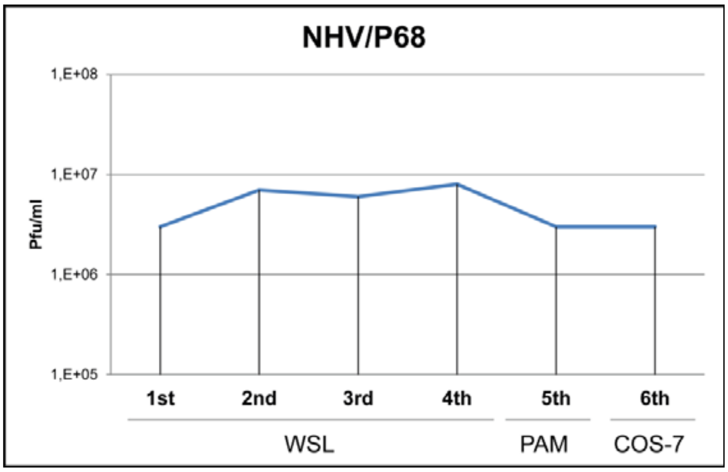

Supplementary Figure S6: Analysis of ASFV infection in PAM after several passages by WSL cells. ASFV NHV/P68 was passed five or ten times consecutively by WSL cells and then PAM were infected with MOI=1. The percentage of infected cells where the virus is able to replicate was analysed by FACS with a specific Ab against the viral protein p72 (17LD3) following of an anti-mouse IgGs Alexa Fluor-488. The graphics represent the percentage of positive cells (n = 2; mean ±S.D.).

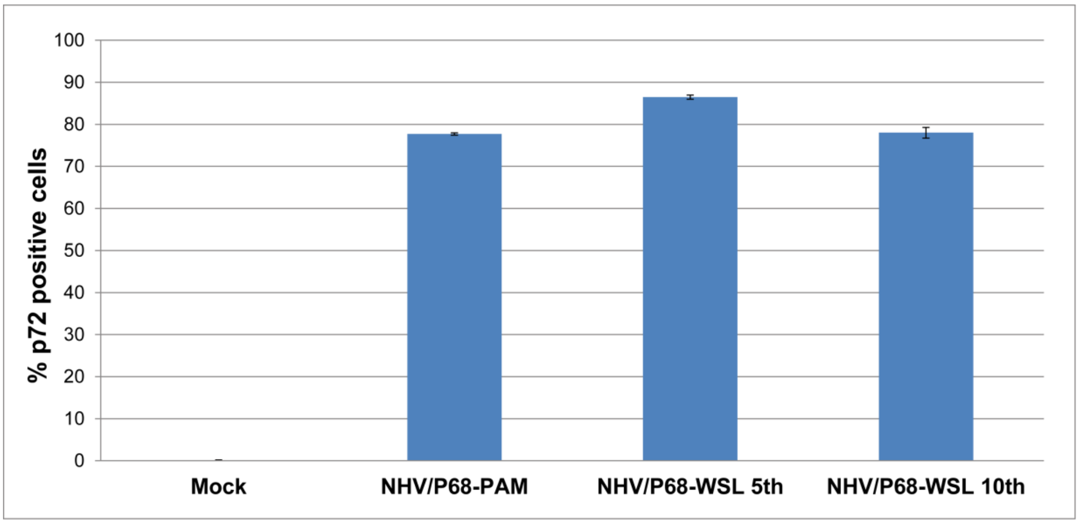

Supplementary Figure S7: Survival rate and clinical score of pigs. Graphic shows the survival rate and clinical score overlapped obtained after the inoculation of pigs with the NHV/P68-WSL ASFV strain passaged ten times in WSL. Pigs were inoculated with either 10^7 TCDI50/ml (red) or 10^5 TCDI50/ml (blue).

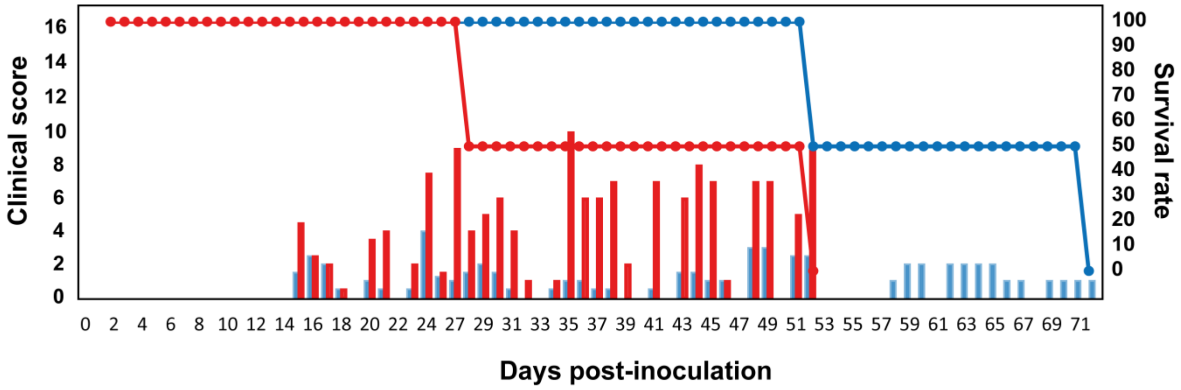

Supplementary Figure S8: Viremia and ASF-specific antibody detection in sera. Virus presence was determined by real time PCR **(a)** and antibody titer was determined by IPT **(b)** in the group of the inoculated animals using either 10^7 TCDI50/ml (pig AT3 and pig AT4) or 10^5 TCDI50/ml (pig AT1 and pig AT2).

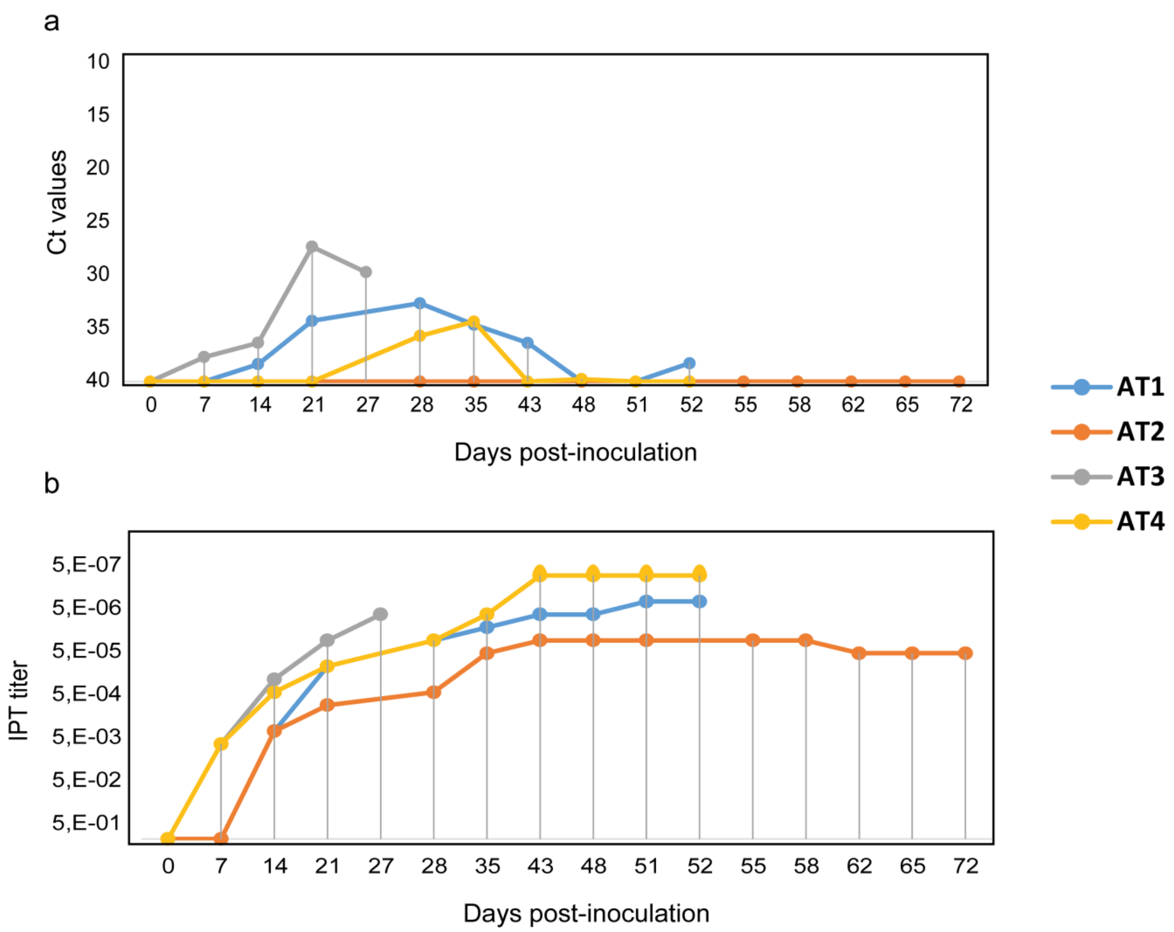

Supplementary Table S1: ASF virus detection in tissues collected from the NHV inoculated animals

Tissue identification	*Pig AT3d27pi*			*Pig AT1 d52pi*			*Pig AT4 d52pi*			*Pig AT2 d72pi*		
	PCR		$VI^{(1)}$	PCR		*VI*	PCR		*VI*	PCR		*VI*
	Ct	*Result*		*Ct*	*Result*		*Ct*	*Result*		*Ct*	*Result*	
Submandibular LN*	22.74	**POS**	**POS**	No Ct	NEG	-	No Ct	NEG	-	No Ct	NEG	-
Retropharyngeal LN	Ns	-	-	36.66	**POS**	NEG	33.87	**POS**	**POS**	No Ct	NEG	-
Mediastinal LN	34.58	**POS**	NEG	No Ct	NEG	-	No Ct	NEG	-	No Ct	NEG	-
Gastro-hepatic LN	31.85	**POS**	**POS**	No Ct	NEG	-	No Ct	NEG	-	No Ct	NEG	--
Renal LN	29.08	**POS**	**POS**	No Ct	NEG	-	No Ct	NEG	-	No Ct	NEG	-
Splenic LN	Ns	-	-	36.5	**POS**	NEG	37.82	**POS**	NEG	No Ct	NEG	-
Mesenteric LN	28.31	**POS**	**POS**	No Ct	NEG	-	No Ct	NEG	-	No Ct	NEG	-
Popliteal LN	24.12	**POS**	**POS**	37.03	**POS**	NEG	No Ct	NEG	-	No Ct	NEG	-
Inguinal LN	24.69	**POS**	**POS**	37.87	**POS**	NEG	36.95	**POS**	NEG	No Ct	NEG	-
Tonsil	36.21	**POS**	NEG	No Ct	NEG	-	36.58	**POS**	NEG	No Ct	NEG	-
Heart	37.26	**POS**	NEG	No Ct	NEG	-	No Ct	NEG	-	No Ct	NEG	-
Lung	30.84	**POS**	**POS**	38.19	**POS**	NEG	30.98	**POS**	**POS**	No Ct	NEG	-
Liver	32.72	**POS**	NEG	No Ct	NEG	--	No Ct	NEG	-	No Ct	NEG	-
Spleen	32.17	**POS**	NEG	No Ct	NEG	-	No Ct	NEG	-	No Ct	NEG	-
Kidney	32.86	**POS**	NEG	No Ct	NEG	-	No Ct	NEG	-	No Ct	NEG	-
Bone marrow	32.71	**POS**	NEG	No Ct	NEG	-	38.02	**POS**	NEG	No Ct	NEG	-
Back right IA**	30.07	**POS**	NEG	No Ct	NEG	-	No Ct	NEG	-	37.03	**POS**	NEG
Back left IA	29.93	**POS**	**POS**	No Ct	NEG	-	No Ct	NEG	-	No Ct	NEG	-
Front right IA	30.07	**POS**	NEG	31.34	**POS**	**POS**	No Ct	NEG	-	No Ct	NEG	-
Front left IA	18.85	**POS**	**POS**	No Ct	NEG	-	No Ct	NEG	-	37.96	**POS**	NEG
Skin lesions (joint)	Ns	-	-	Ns	-	-	28.17	**POS**	**POS**	21.96	**POS**	**POS**

**Lymph node; ** intra-articular tissues; (ns = no sample)*
(1) virus isolation result after three passages in PBM

Supplementary Table S2: Antibodies used by FACS in the present study

**Antigen**	**Antibody**	**Isotype**	**Source**
Pig CD14	CD14-FITC; MIL-2	IgG2b	Bio-Rad MCA1218F
Pig CD163	2A10	IgG1	Dr. Javier Domínguez
Pig CD169	1F1/CR4	-	Dr. Javier Domínguez
Pig SLAII	1F12	IgG2b	Dr. Javier Domínguez
Pig SLAI	4B7/8	IgG2a	Dr. Javier Domínguez
Pig SWC3	BA1C11	IgG1	Dr. Javier Domínguez
ASFV p72	17LD3	IgG2b	Ingenasa
Mouse IgG	Alexa Fluor-488	panIgG	Thermo Fisher
Mouse IgG	Alexa fluor-647	panIgG	Thermo Fisher
Human IgG2b	IgG2b-FITC	IgG2b	BD Bioscience
